# RetroScan: An Easy-to-Use Pipeline for Retrocopy Annotation and Visualization

**DOI:** 10.3389/fgene.2021.719204

**Published:** 2021-08-16

**Authors:** Zhaoyuan Wei, Jiahe Sun, Qinhui Li, Ting Yao, Haiyue Zeng, Yi Wang

**Affiliations:** ^1^State Key Laboratory of Silkworm Genome Biology, Biological Science Research Center, Southwest University, Chongqing, China; ^2^Biological Science Research Center, Southwest University, Chongqing, China

**Keywords:** retrocopy, pipeline, evolution, visualization, genome

## Abstract

Retrocopies, which are considered “junk genes,” are occasionally formed via the insertion of reverse-transcribed mRNAs at new positions in the genome. However, an increasing number of recent studies have shown that some retrocopies exhibit new biological functions and may contribute to genome evolution. Hence, the identification of retrocopies has become very meaningful for studying gene duplication and new gene generation. Current pipelines identify retrocopies through complex operations using alignment programs and filter scripts in a step-by-step manner. Therefore, there is an urgent need for a simple and convenient retrocopy annotation tool. Here, we report the development of RetroScan, a publicly available and easy-to-use tool for scanning, annotating and displaying retrocopies, consisting of two components: an analysis pipeline and a visual interface. The pipeline integrates a series of bioinformatics software programs and scripts for identifying retrocopies in just one line of command. Compared with previous methods, RetroScan increases accuracy and reduces false-positive results. We also provide a Shiny app for visualization. It displays information on retrocopies and their parental genes that can be used for the study of retrocopy structure and evolution. RetroScan is available at https://github.com/Vicky123wzy/RetroScan.

## Introduction

Gene duplications, which are generated by DNA- or RNA-mediated mechanisms (Innan and Kondrashov, 2010; Sakai et al., 2011), are a major source of the origination of new genes (Long et al., 2003) and play pivotal roles in genome evolution, new biological process origination and functional diversification (Flagel and Wendel, 2009). Retrocopies are a special type of RNA-mediated duplication (Brosius, 1991) in which the reverse transcripts of mRNAs derived from parental genes are occasionally reinserted at an ectopic location in the genome (Long et al., 2003). Retrocopies are new sequence fragments formed by retrotransposition events. Most retrocopies are non-functional due to their insertion at inappropriate sites or a lack of parental gene features such as introns or regulatory elements and are believed to be retropseudogenes (Lynch and Conery, 2000; Navarro and Galante, 2013). Another group of retrocopies may inherit the complete open reading frames (ORFs) of the parental genes or recruit regulatory elements such as promoters, enhancers and coding sequences from flanking regions to generate a functional retrogene (Pan and Zhang, 2009). Furthermore, the fusion of a retrocopy with coding sequences near the insertion site generates a chimeric gene (Betran et al., 2002; Wang et al., 2002). Recent studies have systematically identified a substantial number of retrocopies in the genomes of fruit flies (Bai et al., 2007), *Caenorhabditis elegans* (Schrider et al., 2011), humans (Ohshima et al., 2003; Zhang et al., 2003; Vinckenbosch et al., 2006), zebrafish (Fu et al., 2010), and other mammals (Pan and Zhang, 2009). Some studies have searched for retrocopies in plant genomes, mainly in *Arabidopsis thaliana* (Zhang et al., 2005), rice (Sakai et al., 2011), poplar (Zhu et al., 2009), and green algae (Jąkalski et al., 2016). Moreover, some functions of retrocopies have been verified through experiments; for example, Jingwei functions in the metabolism of recruitment pheromones and juvenile hormones in fruit flies (Long and Langley, 1993; Zhang et al., 2010), and CYP98A8 and CYP98A9 are involved in pollen development in *Arabidopsis thaliana* (Matsuno et al., 2009). Retrocopies not only contribute to the diversity of genome sequences but can also cause rapid and significant changes in the genome by altering genome structures. Therefore, they are an important driving force for the origination of new genes (Carelli et al., 2016) and provide evidence of evolutionary innovations (Navarro and Galante, 2015). With the rapid development of next-generation sequencing technology, many studies have assembled chromosome-level genomes of new species, and a tool for annotating retrocopies at the genome-wide level would help us to fully understand their positions in the genome and the process of their production. Such a tool would be highly significant for studying genome evolution and subsequently analyzing the function of retrocopies (Kaessmann et al., 2009).

Since retrocopies have often lost introns and but are otherwise highly similar to their parental genes, the identification of retrocopies in the whole genome is generally based on the use of protein sequences as templates for sequence alignment. Current retrocopy identification pipelines are based mainly on the TBLASTN, BLAT, and paralog methods (Casola and Betrán, 2017). Most studies of retrocopies are based on the TBLASTN method, which aligns the annotated protein-coding sequences to whole-genome sequences. Candidate hits are determined by alignment with parental genes to determine the numbers of lost introns, point mutations and frameshift mutations using FASTA (Pearson and Lipman, 1988) and GENEWISE (Birney et al., 2004). This method has been used to find retrocopies in humans (Vinckenbosch et al., 2006), *Caenorhabditis elegans* (Abdelsamad and Pecinka, 2014), *Arabidopsis thaliana* (Zhang et al., 2005), rice (Sakai et al., 2011), poplar (Zhu et al., 2009), and green algae (Jąkalski et al., 2016). However, the speed of the TBLASTN method is relatively slow, and scanning a large genome often takes several days or even a few weeks. But Kabza et al. (2014) were the first use LAST to identify retrocopies instead of TBLASTN, which greatly improved the speed of alignment. The use of BLAT to align genomic sequences with cDNA sequences instead of proteins is also a good option. The BLAT method directly estimates the number of missing introns according to the alignment results without additional programs. However, compared with the TBLASTN method, the BLAT method shows lower accuracy, and some positive retrocopies will be ignored. This is not conducive to further evolutionary analysis because the BLAT method cannot get the proteins mutations information between parental genes and retrocopies. Navarro and Galante (2015) used the BLAT method to scan for retrocopies in seven primate genomes, and the PlantRGDB database provides annotations for the retrocopies of 49 plant genomes (Wang, 2017). Moreover, a new method developed by Abdelsamad and Pecinka (2014) divides the annotated genes into two types, intron-free genes and intron-containing genes, and then aligns them using paralogs to identify retrocopies. Compared to the previous two methods, this approach can find more retrocopies in intron-free genes but also produces more false-positive results. It is impossible to find retropseudogenes via the paralog method because it focuses only on annotated genes rather than genome sequences. All of the above methods for identifying retrocopies present some disadvantages. Therefore, there is an urgent need to develop a comprehensive and uncomplicated tool for identifying, annotating and analyzing retrocopies in the genome which could facilitate in-depth research on retrocopies.

In the development of an easy-to-use retrocopy identification pipeline, the following requirements must be met. First, the increasing number of genome sequences generated by high-throughput sequencing technology have brought retrocopy research a new era, so the new pipeline must be suitable for various species, including large-scale genomes. Second, it must be convenient for users to configure and run, requiring few extra operations. Third, it should effectively reduce false-positive results. Finally, all results should be clearly displayed in the form of clear figures. To meet all of these design needs, we developed a convenient and accurate tool, RetroScan,^1^ which is based on the method of aligning protein sequences with genome sequences to recognize retrocopies by integrating multiple software programs and scripts. Next, RetroScan was used to explore the expression, age distribution and functions of the retrocopies. Finally, we constructed a reliable graphical interface to display the results, thus helping researchers to easily obtain information on retrocopies and achieve a deep understanding them.

## Materials and Methods

RetroScan is an easy-to-use tool for retrocopy identification that integrates a series of bioinformatics tools [LAST (Kielbasa et al., 2011), BEDtools (Quinlan and Hall, 2010), ClustalW2 (Larkin et al., 2007), KaKs_Calculator (Wang et al., 2010), HISAT2 (Kim et al., 2015), StringTie (Pertea et al., 2015), SAMtools (Li et al., 2009), and Shiny] and scripts. It scans retrocopies based on alignments between protein-coding genes and whole-genome sequences. This tool can also analyze heterosense substitution and synonymous substitution, compare gene structure between parental genes and retrocopies, and calculate corresponding expression values. Moreover, RetroScan has a user-friendly visualization interface that provides overall statistical information, a retrocopy structure diagram, the non-synonymous/synonymous substitution (Ka/Ks) ratio distribution and the fragments per kilobase per million (FPKM) heatmap using the Shiny package in R.

### Retrocopy Identification

**FIGURE 1:**
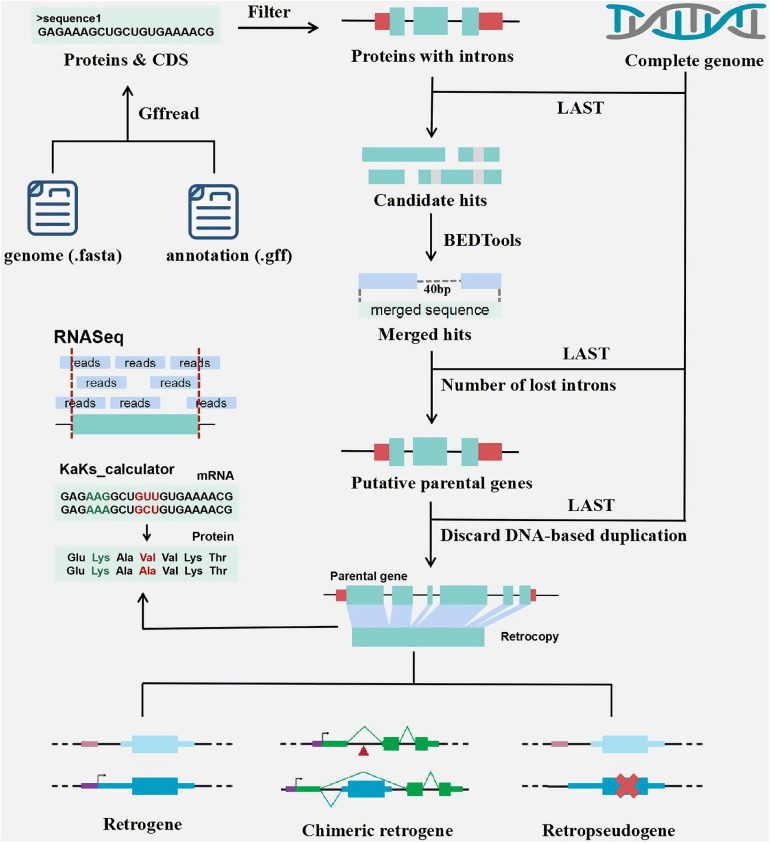
The pipeline of retrocopy annotation.

RetroScan mainly relies on the identification of genomic intronless alignments from mature transcripts (mRNAs) for the reason that retrocopies are processed copies of multiexon proteins. It requires at least two input files (Figure 1): a genome sequence file (FASTA format) and a corresponding annotation file (GFF format), from which it can provide detailed information on retrocopies and parental genes in the genome. If users wish to obtain the expression values of retrocopies, they need to submit additional RNA-Seq data.

According to genome sequences and GFF file (Figure 1), RetroScan first employs the peptide sequences used as queries in similarity searches against complete genome sequences using LAST to identify candidate hits. To avoid duplicate results, the longest transcripts of each gene for alignment are retained for the next step. Multiexon proteins are selected for subsequent analysis because the parental genes must lose at least two introns. According to the alignment results from the previous step, users can set the sequence identity, coverage and alignment length parameters to consider the specific conditions of the species. Multiple alignment hits to the same genomic locus are clustered using BEDTools. When the distance between the hits is less than a certain length, indicating that they are unlikely to be separated by introns, adjacent homology hits are merged using BEDTools. The gap default is 40 bp in RetroScan, but if users want to change this threshold, they should take into consideration that the length of most introns ought to be larger than the threshold.

Next, the merged sequences are aligned back to multiexon proteins using LAST, and the best hits are retained as putative parental genes. Finally, the number of lost introns is estimated to obtain reliable results according to the alignment output. We calculate the position of the introns on the protein sequences according to the annotation file. RetroScan only retains parental genes (excluding the first and last 10 amino acids) that span at least two introns and single-exon retrocopies. We discard any cases involving possible DNA-based duplications by aligning retrocopy sequences back to genome sequences to minimize the number of false-positive results. If a retrocopy shows multiple highly similar sequences in the genome, it will be deleted.

In addition, retrocopies with either premature stop codons or frameshift mutations are defined as retropseudogenes; otherwise, they are defined as intact retrocopies. If one intact retrocopy can recruit novel regulatory elements or new protein-coding exons and evolve into a functional retrogene, it can be defined as a chimeric retrogene. RetroScan is more convenient and easier to use, which integrates multiple softwares and there is no need for the user to call the softwares at each step. Compared with the traditional processes, LAST alignment is faster. We also align the results of retrocopy back to the genome to avoid rertocopy caused by DNA duplication, which effectively reduces false positives.

### Ka/Ks Analysis

The age distribution of the retrocopies (Figure 1) is determined by calculating Ka, Ks and the Ka/Ks ratio between each retrocopy and its parental gene. The coding sequence (CDS) information of the retrocopies and their parental genes based on the annotation file are extracted for Ka/Ks calculation. Then, RetroScan performs multiple alignments between the corresponding protein sequences using ClustalW2. Finally, the Ka, Ks, and Ka/Ks values are calculated using KaKs_calculator_2.0.

### Retrocopy Expression Analysis

Although the sequences of the parental genes and retrocopies are similar, some retrocopies are not expressed, which implies that they have no function. Some retrocopies exhibit expression patterns similar to those of their parental genes and may have similar functions, and some retrocopies exhibit much higher expression values than their parental genes, which means that they may replace the parental gene function. Therefore, analyzing the expression of retrocopies in different tissues and organs is helpful for exploring their functions. As retrocopies show high similarity with their parental genes, the expression values of them might be biased by the lack of RNA-seq reads mapping uniquely to either copy. There are two factors that could possibly cause this. First, it is well known that retrocopies have very low expression and are usually limited to one or a few tissues (Carelli et al., 2016). Secondly, sequences that matched equally well to a given retrogene progenitor were excluded what additionally reduced the number of positive results (Rosikiewicz et al., 2017). To estimate the expression values of retrogenes (Figure 1), RetroScan uses HISAT2, SAMtools, and StringTie to analyze the RNA-Seq data based on retrocopy and parental gene position information, which has the advantages of high accuracy and fast speed. After the reads are mapped to the corresponding annotated sequences using HISAT2, RetroScan converts SAM files into BAM files and sorts them using SAMtools. Finally, StringTie calculates FPKM values, which are helpful for analyzing differential expression. All programs are run with the default settings.

### Visualization

We developed a visual interface that can clearly display retrocopy structure, the ka/ks distribution, expression levels, sequence alignments and statistical figures. We use R to analyze the RetroScan results, while the web pages are mainly built with Shiny and a series of R packages such as ggplot2, UpSetR, ggmsa, VennDiagram, dplyr, DT, shinydashboard, Biostrings, muscle, pheatmap, stringr, shinyjs, RColorBrewer, ape, etc. The interface layout is divided into four parts: summary, retrocopy, KaKs and expression. Users can upload the RetroScan result files generated by RetroScan through the START button on the homepage.

**FIGURE 2:**
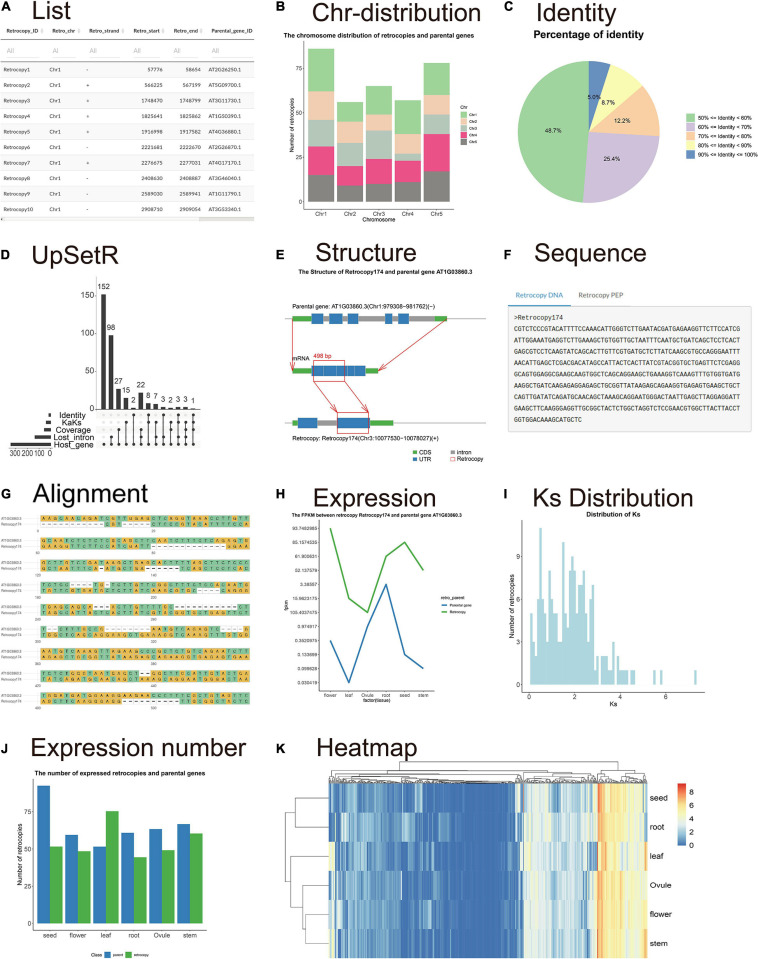
Visualization of the retrocopy results. **(A)** The table contains all information on retrocopies and parental genes. **(B)** The chromosome distribution of retrocopies. **(C)** The percentage of identity. **(D)** The UpSet plot visualizes the intersections of datasets showing an identity ≥ 90%, ≥ 3 lost introns, host genes, Ka/Ks ≤ 0.1 and coverage ≥ 90%. **(E)** The structure figure shows the differences in the gene sequences between the parental genes and retrocopies. **(F)** The retrocopy sequence and the parental gene mRNA and protein sequences. **(G)** Sequence alignment between the retrocopy and parental gene. **(H)** The expression values of retrocopy and parental genes. **(I)** Ks distribution histograms. **(J)** Histogram showing the mean FPKM values of retrocopies (blue bar) and parental genes (brown bar) in all tissues. **(K)** Heatmap showing the expression of all retrocopies.

The “Summary” page shows the RetroScan results and related statistical information which are mainly displayed in the form of tables, histograms, pie charts, line graphs, Venn diagrams, heat maps, and so on. There is a table containing all of the information for retrocopies and their parental genes, including the retrocopy ID, chromosome, start site and end site of the retrocopy; the parental gene ID, identity, coverage, and description; and the host gene ID (Figure 2A). The other parts of the page show seven statistical figures illustrating the chromosome distribution of the parental genes corresponding to the retrocopies on each chromosome (Figure 2B), the distribution of the number of retrocopies of by each parental gene, the retrocopy length distribution, the percentage of identity (Figure 2C), the percentage of coverage and the percentage of retropseudogenes, intact retrocopies and chimeric retrocopies. The static UpSet plot (Conway et al., 2017; Figure 2D) visualizes the intersections of datasets showing an identity ≥ 90%, ≥ 3 lost introns, host genes, a Ka/Ks ≤ 0.1, and coverage ≥ 90% in a matrix layout and introduces aggregates based on groupings and queries. The upper bar graph corresponds to the lower dot matrix graph including the intersections of related datasets.

The “Retrocopy” page includes a search box where users can enter any retrocopy ID. The search result integrates the detailed information, sequence structure, alignment and expression of a certain retrocopy. The structure figure (Figure 2E) shows the structural differences in the gene sequences among the parental genes, retrocopies and host genes so that users can clearly understand the formation of retrocopies from parental genes. The sequence section contains the sequences of the retrocopy gene and protein sequences (Figure 2F). The alignment section shows the sequence alignment between the retrocopy and the parental gene to allow users to identify the differences in bases (Figure 2G). The expression patterns in different developmental stages and tissues could be used as a basis for judging whether a retrocopy has a biological function and whether there is functional correlation between the retrocopy and its parental gene. The page displays the expression values in a line chart in which two lines represent the expression of the retrocopy and the parental gene (Figure 2H).

A Ka/Ks table and four statistical figures are provided to investigate the origin and evolution of retrocopies on the “KaKs” page. Users can view the table of Ka, Ks, and Ka/Ks values and set reasonable thresholds for filtering retrocopies. The age distribution is shown with a Ks histogram and is estimated by comparing the protein sequences of the parental genes and retrocopies (Figure 2I). Another Ks histogram shows the Ks distribution in three categories: retropseudogenes, intact retrocopies and chimeric retrocopies.

The expression page provides information on estimated retrocopy expression. The table shows the accurate FPKM values of the retrocopies and their parental genes. The histogram shows the mean FPKM values for each tissue (Figure 2J). Moreover, the heatmap shows the expression of all retrocopies (Figure 2K). The heatmap clearly shows the tissues in which retrocopies are highly expressed or not expressed, so that user can explore the function of retrocopies and whether their expression shows an organizational preference.

Users can filter the data based on any table column on each page and can directly search for keywords in the search box above the tables. All image colors and text sizes can be adjusted according to users’ needs. All information tables and figures can be downloaded by clicking the download tabs.

## Results

### Test

RetroScan is suitable for species with available scaffold-level or chromosome-level genome assemblies and detailed annotation information. If users upload the relevant RNA-Seq data, they can further explore the expression values of retrocopies. A well-developed retrocopy annotation tool requires tests to examine its accuracy and improve its applicability. Here, we selected six eukaryotic species for verification, including two vertebrates [*Homo sapiens* (Falconer et al., 2012), *Danio rerio* (Howe et al., 2013)], two plants [*Arabidopsis thaliana* (Theologis et al., 2000), *Oryza sativa* (Sasaki and International Rice Genome Sequencing Project, 2005)] and two insects [*Drosophila melanogaster* (Adams et al., 2000), *Aedes aegypti* (Nene et al., 2007)]. The data were all downloaded from NCBI (Supplementary Table 1). In addition, we also tested species genomes from databases such as JGI (Phytozome), Ensembl and FlyBase (Supplementary Table 2). In our tests, RetroScan performed well and was suitable for genomic data of various databases. The running time and results of RetroScan are listed in Table 1. We ran RetroScan by entering the genome sequence files and corresponding annotation files. For evaluation, the programs were run on a dedicated Linux machine with Ubuntu18.04 running no other job, using the GNU time command to obtain real time. The machine had 16 GB of physical RAM and a six core Intel i7 CPU. We set all parameters to the default settings (thread = 1, identity ≥ 50%, coverage_rate ≥ 50%, coverage_len ≥ 50 aa, intron_loss_num ≥ 2, gap_len ≥ 40 bp, parent_loss_intron_len ≥ 60 bp, retro_one_exon_len ≤ 30, kaksmethod = NG). The size of the genomes ranged from 121 M to 3.3 G, and the number of retrocopy results reached 7048. The size of the genome, the number of annotated proteins and the proportion of repeated sequences have the greatest impact on the running time.

**TABLE 1:** RetroScan results for retrocopies in *Homo sapiens*, *Danio rerio*, *Arabidopsis thaliana*, *Oryza sativa*, *Drosophila melanogaster*, and *Aedes aegypti*.

Species	Genome size	Protein number	Time	Retrocopy number
*Homo sapiens*	3.3 G	1,302,060	768 min	7,048
*Danio rerio*	1.7 G	659,618	256 min	449
*Drosophila melanogaster*	145.7 M	336,015	11 min	221
*Aedes aegypti*	1.3 G	330,718	61 min	410
*Arabidopsis thaliana*	121.2 M	259,756	13 min	343
*Oryza sativa*	387.6 M	189,861	27 min	661

### Comparison With Previous Studies

**FIGURE 3:**
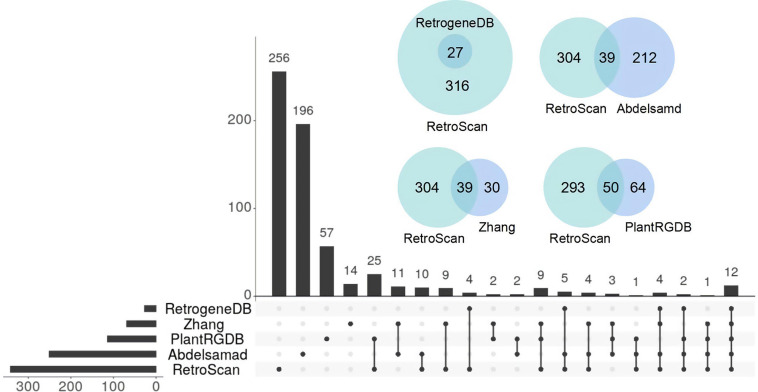
Comparison of *Arabidopsis thaliana* retrocopies identified by RetroScan and four other methods.

**FIGURE 4:**
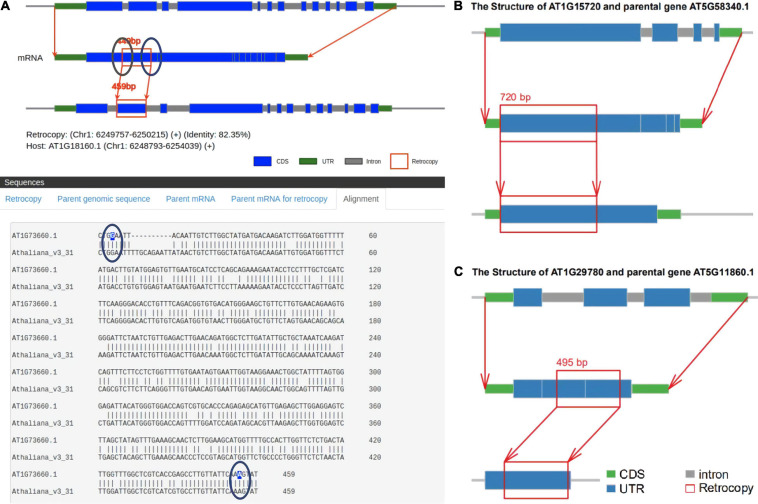
False-positive retrocopies. **(A)** The sites of lost introns are located in the marginal area of the retrocopies in PlantRGDB. **(B)** The parental genes identified by Zhang do not lose any introns. **(C)** The parental genes identified by Abdelsamad lose only one intron.

There is a lack of a uniform definition of retrocopy identity. The criteria for judging retrocopies are based mainly on the core definition that the sequences of retrocopies and their parental genes are highly similar but the parental genes lose multiple introns. Current retrocopy identification pipelines are based on the TBLASTN, BLAT, and paralog methods, and we selected representative studies in these pipelines to compare with RetroScan: RetrogeneDB (Rosikiewicz et al., 2017) for TBLASTN, PlantRGDB (Wang, 2017) for BLAT and the study of Abdelsamad and Pecinka (2014) and Zhang et al. (2005) for paralog (Supplementary Table 3). The results between these methods vary greatly, so we used *Arabidopsis thaliana* as an example to explain the reasons for these differences. RetroScan includes 343 retrocopies, RetrogeneDB includes 27, PlantRGDB includes 114 (duplicates have been removed), Zhang includes 69 and Abdelsamad includes 251. To compare other results with those of RetroScan, we considered any two retrocopies that overlapped at the same genomic position in which the overlap region was more than 50% of their sequence length to be the same retrocopy. An UpSet plot was generated to represent the intersections between five datasets (Figure 3). The total number of retrocopies in all studies was 627. Among the RetroScan retrocopies, 87 were shared with retrocopies from other pipelines, and 256 were novel (Figure 3). The 256 novel retrocopies consisted partly of retropseudogenes, which were mainly distributed in non-coding regions. Other novel retrocopies were newly discovered retrocopies that were ignored by the other four pipelines. We observed that all of the RetrogeneDB retrocopies overlapped with the RetroScan results because that study applied a similar pipeline to directly align protein-coding sequences with genome sequences using LAST. However, RetrogeneDB involved more stringent criteria (e.g., regarding alignment length, identity and coverage), and few retrocopies could be found in non-coding regions. RetroScan and PlantRGDB showed only 50 overlapping results, as PlantRGDB used the BLAT tool to identify retrocopies in plants. The BLAT method is not as accurate as BLASTN and will result in the loss of some positive results. The parental genes identified by the BLAT method do indeed lose multiple introns, but the sites of lost introns are located in the marginal area of the retrocopies, which are easily excluded in RetroScan (Figure 4A). Abdelsamad and Zhang developed a new method for identifying retrocopies. The method mainly compares intron-free genes and intron-genes with paralogs to find retrocopies. The paralog method can find more retrocopies in intron-free genes than the previous two methods but also produces more false-positive results. Therefore, only 39 overlapping results were observed with the results of this method. Moreover, it cannot find retropseudogenes because it only uses annotated genes rather than genomic sequences. A portion of the retrocopies identified by the paralog method were found in the ortholog clusters shared with other species, such as rice. Another possibility is that parental genes with multiple exons do not lose any introns (Figure 4B) or lose only one intron (Figure 4C) in the region corresponding to the retrocopies. RetroScan can solve most of the above problems. Because two alignments are performed, mapping proteins to genome sequences and confirming lost introns, RetroScan guarantees that the results are accurate and reliable.

## Discussion

Retrocopies are fragments of genomic sequences which are highly similar to protein coding genes. They were considered as non-functional pseudogenes at some time in the past. Approaches established to identify pseudogenes include PseudoPipe (Zheng and Gerstein, 2006), HAVANA method (Searle et al., 2004), PseudoFinder (Chen et al., 2011), RetroFinder (Zheng et al., 2007), GIS-PET method (Ng et al., 2005), and consensus method (Zheng et al., 2007). These methods were developed by different teams, which mainly use alignment tools such as Blast, Blastz, and Blat to align DNA, protein, cDNA, and mRNA sequences and then accord to homology, intron-exon structure, existence of stop codons or frameshifts and so on to judge whether it is a pseudogene. However, not all retrocopies are pseudogenes, which are formed by retrotransposition and partly play some regulatory or other important roles in genome. Therefore, based on the identification of pseudogenes, researchers have developed new identification methods specifically for retrocopies by exhaustively aligning of genomic sequences against all possible parental genes. But different prediction methods often result in different numbers or sets of retrocopies because each researcher uses different criteria for identification.

Here, we draw up the criteria for judging retrocopies by RetroScan, which is a promising software developed to scan, annotate and display retrocopies. Regarding the coverage, similarity, the number of lost introns and other parameters between the parental genes and retrocopies, users can set according to the species situation. Compared to previous approaches, our new computational analysis tool shows increased accuracy and speed and is more convenient to use, especially when processing species with large-scale genomes. RetroScan is faster than the BLAT method and produces fewer false positives, similar to the paralog method. We used six species data to compare the results of RetroScan and three classic pipelines. Compared the sequence structure of retrocopies with parental genes, we found that RetroScan had the lowest false positives. At the same time, we ensure that the final results have nothing to do with DNA duplication by comparing the results back to the genome and deleting retrocopies with a large number of duplicates. It involves only one step and requires at least two input files (genome sequence file and annotation file). If RNA-Seq data are provided, it can further calculate the expression values of retrocopies. We used multiple sets of model species genomes for testing, and the results proved that RetroScan is effective for the identification of retrocopies. In addition, our study is the first to provide a user-friendly visual interface that displays results, including information on retrocopies, Ka/Ks values, retrocopy structure and expression. Our approach shows great potential for retrocopy identification and will make an important contribution to evolutionary research, providing a powerful tool for promoting research on the duplication of genes and the origination of new genes and new functions.

Unlike RetroScan that identifies retrocopies of a single species, there are studies that focus on the genetic variations between groups. Schrider et al. (2013) describe a computational approach leveraging next-generation sequence data to detect gene copy-number variants caused by retrotransposition (retroCNVs), and find that these variants account for a substantial number of gene copy-number differences between individuals, and that gene retrotransposition may often result in both deleterious and beneficial mutations. Miller et al. (2021) exploit sideRETRO, a pipeline dedicated to detecting retroCNVs in whole-genome sequencing data and revealing their insertion sites, zygosity and genomic context and classifying them as somatic or polymorphic events. These tools focus on identifying the CNVs of retrocopy in the population, while RetroScan contributes greatly to research on retrocopies in individual organisms, which is of great significance for establishing a foundation for the future analysis of retroCNVs between subgroups.

In summary, RetroScan is a comprehensive, efficient and one-step retrocopy identification tool developed for users. We firmly believe that RetroScan will be useful for further comparative and evolutionary studies.

## Data Availability Statement

RetroScan is available at https://github.com/Vicky123wzy/RetroScan and can be installed directly by Conda. Users can also download the source code from GitHub, install related software and manually configure RetroScan. The data used in this study were retrieved from NCBI (https://www.ncbi.nlm.nih.gov/). Further inquiries can be directed to the corresponding author/s.

## Author Contributions

ZW developed the tool and drafted the manuscript. JS packaged, uploaded, and tested the tool. QL and TY participated in data testing. HZ revised the manuscript. YW designed and supervised the study and reviewed the manuscript. All authors contributed to the article and approved the submitted version.

## Conflict of Interest

The authors declare that the research was conducted in the absence of any commercial or financial relationships that could be construed as a potential conflict of interest.

## Publisher’s Note

All claims expressed in this article are solely those of the authors and do not necessarily represent those of their affiliated organizations, or those of the publisher, the editors and the reviewers. Any product that may be evaluated in this article, or claim that may be made by its manufacturer, is not guaranteed or endorsed by the publisher.

## Footnotes

1
https://github.com/Vicky123wzyw/RetroScan
