# Supplementary material for: RetroScan: An Easy-to-Use Pipeline for Retrocopy Annotation and Visualization
Source: Front Genet. 2021 Aug 16;12:719204. doi: 10.3389/fgene.2021.719204 (PMC8415311; doi:10.3389/fgene.2021.719204)
Supplement: Supplementary file 1 [file Data_Sheet_1.docx]

Supplementary Material

# Supplementary Table 1. Information of test data of NCBI.

| Species | Download | Bioproject |
| --- | --- | --- |
| *Homo sapiens* | ftp://ftp.ncbi.nlm.nih.gov/genomes/all/GCF/000/001/405/GCF_000001405.39_GRCh38.p13/GCF_000001405.39_GRCh38.p13_genomic.fna.gz | PRJNA745248 |
| *Danio rerio* | https://ftp.ncbi.nlm.nih.gov/genomes/all/GCF/000/002/035/GCF_000002035.6_GRCz11/GCF_000002035.6_GRCz11_genomic.fna.gz | PRJNA11776 |
| *Drosophila melanogaster* | https://ftp.ncbi.nlm.nih.gov/genomes/all/GCF/000/001/215/GCF_000001215.4_Release_6_plus_ISO1_MT/GCF_000001215.4_Release_6_plus_ISO1_MT_genomic.fna.gz | PRJNA719972 |
| *Aedes aegypti* | ftp://ftp.ncbi.nlm.nih.gov/genomes/all/GCF/002/204/515/GCF_002204515.2_AaegL5.0/GCF_002204515.2_AaegL5.0_genomic.fna.gz | PRJNA19731 |
| *Arabidopsis thaliana* | ftp://ftp.ncbi.nlm.nih.gov/genomes/all/GCF/000/001/735/GCF_000001735.4_TAIR10.1/GCF_000001735.4_TAIR10.1_genomic.fna.gz | PRJNA727954 |
| *Oryza sativa* | https://ftp.ncbi.nlm.nih.gov/genomes/all/GCF/000/005/425/GCF_000005425.2_Build_4.0/GCF_000005425.2_Build_4.0_genomic.fna.gz | PRJNA732795 |

# Supplementary Table 2. Test data from other databases.

| Species | Genome size | Protein number | Time | Retrocopy number | Database | Download |
| --- | --- | --- | --- | --- | --- | --- |
| *Citrus sinensis* | 324.9 M | 15060 | 12 min | 417 | Phytozome | https://phytozome.jgi.doe.gov/pz/portal.html |
| *Prunus persica* | 230.1 M | 17152 | 12 min | 519 | Phytozome | https://phytozome.jgi.doe.gov/pz/portal.html |
| *Drosophila simulans* | 128.0 M | 7950 | 6 min | 310 | Flybase | http://flybase.org/ |
| *Poecilia formosa* | 761.7 M | 20698 | 19 min | 1452 | Emsembl | [http://asia.ensembl.org/Poecilia_formosa](http://asia.ensembl.org/Poecilia_formosa/Info/Index) |
| *Scleropages formosus* | 797.6 M | 20804 | 69 min | 2866 | Emsembl | [http://asia.ensembl.org/Scleropages_formosus](http://asia.ensembl.org/Scleropages_formosus/Info/Index) |

# Supplementary Table 3. Comparison of the retrocopies results among RetroScan, TBLASTN, BLAT and paralog.

| Species | RetroScan | TBLASTN (RetrogeneDB) | BLAT (PlantRGDB) | Paralog (Abdelsamad et al.) |
| --- | --- | --- | --- | --- |
| *Homo sapiens* | 7048 | 4611 | 4931 | 4488 |
| *Danio rerio* | 449 | 34 | 1343 | 866 |
| *Drosophila melanogaster* | 221 | 83 | 352 | 344 |
| *Aedes aegypti* | 410 | NA | 611 | 256 |
| *Arabidopsis thaliana* | 343 | 27 | 114 | 251 |
| *Oryza sativa* | 661 | 80 | 379 | 398 |
